# Vanishing Lung Syndrome in a Dog: Giant Pneumatocele or Giant Pulmonary Bulla Mimicking Tension Pneumothorax—First Report

**DOI:** 10.3390/vetsci12050501

**Published:** 2025-05-20

**Authors:** Jack-Yves Deschamps, Nour Abboud, Pierre Penaud, Françoise A. Roux

**Affiliations:** Emergency and Critical Care Unit, Oniris VetAgro Bio, Nantes-Atlantic College of Veterinary Medicine, Food Science and Engineering, La Chantrerie, CEDEX 03, 44307 Nantes, France; nour.abboud@oniris-nantes.fr (N.A.); pierre.penaud@oniris-nantes.fr (P.P.); francoise.roux@oniris-nantes.fr (F.A.R.)

**Keywords:** pneumatocele, pulmonary bulla, giant bulla, vanishing lung syndrome, spontaneous pneumothorax, dog

## Abstract

A 6-month-old neutered male Belgian Malinois dog living in a kennel was presented to a veterinary emergency service for the management of severe respiratory distress that had developed within the past 24 h. Thoracic radiographs performed by a referring veterinarian showed abnormalities identified as a pneumothorax. Upon admission to the emergency service, the striking anomalies turned out to be a large intrathoracic air-filled cavity and countless smaller ones causing mechanical compression of the adjacent pulmonary parenchyma and mimicking tension pneumothorax. Emergency management included thoracocentesis followed by placement of a thoracostomy tube. The dog exhibited rapid clinical improvement and recovered completely within a few days, without requiring surgical intervention. Serial follow-up radiographs showed progressive and complete resolution of all lesions. Based on the complete resolution without resection, the main lesion—initially interpreted as a giant pulmonary bulla—was ultimately considered consistent with an acquired pneumatocele. To the authors’ knowledge, this is the first report in veterinary medicine of a vanishing lung syndrome presentation in a dog.

## Simple Summary

This article presents the case of a 6-month-old Belgian Malinois dog admitted for severe respiratory distress evolving over 24 h. Thoracic radiographs revealed a large intrathoracic air-filled cavity, initially interpreted as a giant pulmonary bulla which appeared responsible for compressive pulmonary collapse in the absence of spontaneous pneumothorax. This presentation closely resembles the vanishing lung syndrome described in human medicine. Emergency thoracocentesis followed by thoracostomy tube placement led to rapid clinical improvement, without the need for surgical intervention. The complete resolution of the lesions under conservative treatment led to a retrospective reassessment, considering the possibility that it was not a giant bulla but perhaps a giant pneumatocele.

## 1. Introduction

Air-filled cavities in the lungs—namely blebs, bullae, and pneumatoceles—represent distinct pathological entities. Blebs refer to collections of air located beneath the visceral pleura, on the lung surface [1]. Bullae are abnormal air spaces within the lung parenchyma, formed by the destruction of alveolar walls [1]. Pneumatoceles are acquired thin-walled air-filled cavities, secondary to trauma, barotrauma or infection, and are rarely reported in veterinary medicine [2,3].

These air-filled cavities typically remain asymptomatic until rupture, at which point they may cause pneumothorax, compromising lung expansion and, consequently, gas exchange. Mechanical compression of the lung parenchyma may lead to pulmonary collapse, closely resembling that observed in traumatic pneumothorax. This manifests as restrictive respiratory distress of varying severity, potentially associated with paradoxical respiration, meaning asynchronous thoracic and abdominal movements, symptoms that are common to other pleural conditions [4].

Pneumothorax is defined as the presence of free air in the pleural space, which is the space located between the visceral pleura (covering thoracic organs such as the lungs) and the parietal pleura (lining the thoracic wall, diaphragm, and mediastinum). This virtual space normally contains only a small amount of fluid that facilitates the sliding of lung lobes against each other and the thoracic wall. Pneumothorax results from the loss of pleural integrity. In dogs, most pneumothoraces are of traumatic origin, resulting from road traffic accidents, hunting incidents, falls, or iatrogenic perforations. Spontaneous pneumothorax, occurring without trauma, is much rarer [5] and is generally caused by the rupture of blebs or bullae [1].

This article describes the case of a dog with severe respiratory distress associated with a giant air-filled cavity initially interpreted as a giant bulla in the absence of pneumothorax. Retrospective analysis of the clinical course and imaging findings led to reconsideration of the initial diagnosis, with an alternative interpretation—a pneumatocele—discussed in the dedicated section.

## 2. Case Presentation

A 6-month-old neutered male Belgian Malinois puppy, weighing only 13.5 kg and properly vaccinated and dewormed, was referred to the emergency service of our institution (Oniris, National Veterinary School of Nantes, France) for severe respiratory distress evolving over the previous 24 h.

### 2.1. History

The dog had lived in a shelter since birth, sharing a cage with two other dogs, including its littermate sister. Five days before presentation (on Day -5), the dog was transferred from the shelter to a rescue center in preparation for adoption. On Day -3, two days after arriving at the rescue center, the dog exhibited prostration and a lack of appetite. On Day -1, severe respiratory distress appeared, prompting a consultation with a veterinarian, who diagnosed pneumothorax and hospitalized the dog for observation. No cough was observed that may have suggested canine infectious respiratory disease complex (CIRDC). A hematologic examination revealed leukocytosis (27.9 × 10⁹/L; RI: 6–17) associated with marked neutrophilia (25.1 × 10⁹/L; RI: 3–11.5), suggestive of an acute inflammatory or stress-related response. The following morning (Day 0), due to the severity of the respiratory distress, the dog was referred to our institution.

### 2.2. Clinical Examination

On admission, the dog was conscious but depressed, alternating between orthopnea in a sitting position and sternal recumbency. It exhibited severe restrictive respiratory distress, characterized by an increased respiratory rate (50 breaths per minute) with rapid and shallow inspirations. This respiratory distress was associated with paradoxical breathing. The mucous membranes appeared slightly cyanotic, and oxygen saturation (SpO_2_) was 94% (reference values > 96%). Pulmonary and cardiac auscultation did not reveal any additional or attenuated sounds.

### 2.3. Radiographic Findings

**Figure 1:**
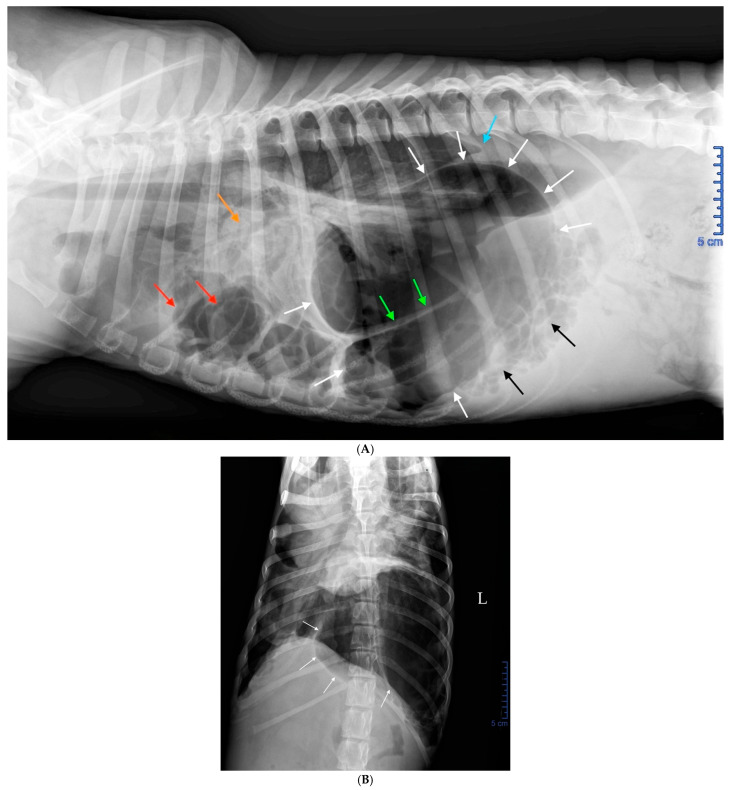
Right lateral (**A**) and dorsoventral (**B**) thoracic radiographs taken by the referring veterinarian the day before admission to the emergency service. In the lateral view (**A**), a giant intrathoracic air-filled cavity (14 cm × 10 cm), delineated by a thin wall (white arrows) and apparently compartmentalized (green arrows), extended from the heart base to the diaphragm, sternum, and lumbar region. Numerous small adjacent cavities were present in contact with the diaphragm (black arrows). Medium-sized air-filled cavities were observed ventrally and cranially (red arrows). The cardiac silhouette was not visible. The pulmonary fields at the level of the cranial lobar bronchi appeared atelectatic (orange arrow), suggesting compressive pulmonary collapse. A mild pleural effusion was present, as evidenced by the retraction of the caudodorsal lung lobes (blue arrow). At this stage, no free air was detected in the pleural space, ruling out pneumothorax. In the dorsoventral view (**B**), although less striking than the lateral view, a large air-filled cavity occupied the caudal part of the left hemithorax, displacing the cardiac silhouette to the right side and caudal displacement of the left diaphragmatic crus mimicking a tension pneumothorax.

The thoracic radiographs performed the previous day by the referring veterinarian revealed spectacular images; however, they did not correspond to a lesion that can be strictly defined as pneumothorax (Figure 1A,B).

### 2.4. Diagnosis

At this stage, the diagnosis was compressive pulmonary collapse caused by mechanical compression of the lungs by a large intrathoracic air-filled cavity, interpreted as a giant bulla, along with numerous smaller accessory air-filled cavities, even before any rupture had occurred. The clinical presentation was attributed to a Hering–Breuer deflation reflex, triggered by insufficient lung expansion secondary to mechanical compression.

### 2.5. Emergency Treatment

Initial treatment aimed to restore pulmonary compliance via emergency thoracocentesis, performed on the conscious dog using a butterfly catheter connected to a three-way stopcock and a large syringe, even before confirmatory radiographs were obtained. Multiple thoracic aspirations were performed on both sides until no more air could be retrieved. A total of 320 milliliters of air was removed during this procedure. The dog’s breathing progressively improved, and oxygen saturation reached approximately 100%. Simultaneously, the dog received flow-by oxygen therapy. Butorphanol was administered intramuscularly (0.3 mg/kg) to provide sedation without causing respiratory depression.

### 2.6. Etiological Hypotheses

Given the dog’s young age and living conditions, the main etiological hypotheses considered at this stage were congenital bullous emphysema or bullous emphysema secondary to bacterial pneumonia, notably CIRDC, or parasitic pneumonia.

### 2.7. Additional Examinations

Thoracic Point-of-Care Ultrasound (POCUS) performed immediately after thoracocentesis confirmed the presence of a mild pleural effusion in the caudodorsal region of the left hemithorax as well as around the heart. Cardiac POCUS showed a left atrium-to-aorta ratio (LA/Ao) of 1.3, ruling out left atrial enlargement. Abdominal POCUS was unremarkable.

Blood gas analysis revealed a pH of 7.41, PaCO_2_ of 29 mmHg (RI: 32–49), and HCO_3_^−^ of 18 mmol/L (RI: 20–26). These values may reflect either a primary respiratory alkalosis with appropriate metabolic compensation, or a mixed acid-base disorder with opposing effects resulting in a normal pH. Hypocapnia was attributed to hyperventilation.

No other biochemical, hematological, or electrolyte abnormalities were present. Blood pressure remained within normal values. Delayed urinalysis was normal.

A fecal Baermann test for *Angiostrongylus vasorum* larvae was negative, as was immunochromatographic antigen testing. Pending these results, empirical treatment with fenbendazole (50 mg/kg PO SID) was initiated and continued for the first five days of hospitalization.

### 2.8. Follow-Up Radiographs

**Figure 2:**
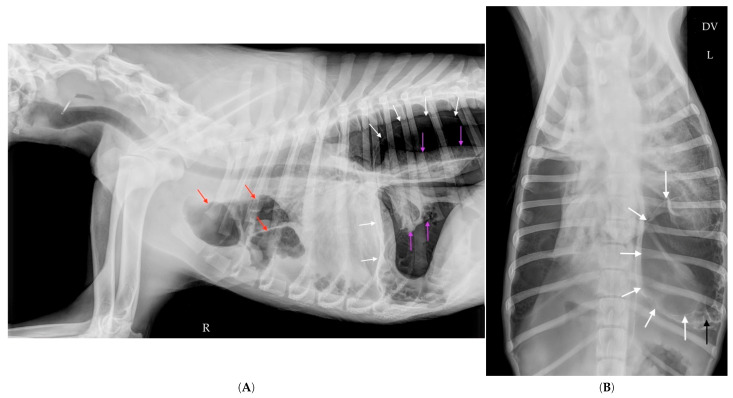
Right lateral (**A**) and dorsoventral (**B**) thoracic radiographs taken on Day 0, one hour after needle thoracocentesis performed upon admission. In the lateral view (**A**), the large intrathoracic air-filled cavity (white arrows) previously seen remains visible but appears reduced in volume. It still occupies the caudodorsal aspect of the thoracic cavity, extending from the diaphragm to the region dorsal to the cardiac silhouette. Cranial and ventral to the heart, multiple medium-sized air-filled cavities (red arrows) persist and are surrounded by a mild pleural effusion. A rounded opacity near the thoracic inlet suggests suprasternal lymphadenopathy, although this finding remains equivocal. The caudal pulmonary lobes appear atelectatic (purple arrows), consistent with compressive pulmonary collapse secondary to the large air-filled cavity. The cardiac silhouette, now clearly visible, is not elevated from the sternum, arguing against the presence of a significant pneumothorax. In the dorsoventral view (**B**), the gas-filled cavity is localized to the left hemithorax, supported by the position of the gastric bubble. Although reduced, it still occupies approximately one-third of the left hemithorax. Numerous satellite air-filled cavities remain visible around the main cavity (black arrow), forming a complex cavitary network.

Follow-up radiographs taken one hour after thoracocentesis showed the persistence of a large air-filled cavity located in the caudal part of the left hemithorax (white arrows), albeit reduced in size compared to the initial images from the day before (Figure 2A,B). The thoracocentesis needle likely entered the giant cavity during one of the aspirations, thereby reducing its volume.

### 2.9. Thoracostomy Tube Placement

To complete the removal of air from the giant air-filled cavity and to avoid repeated thoracocenteses, a thoracostomy tube was placed through the left 10th intercostal space using a modified Seldinger technique under ultrashort anesthesia (propofol alone, no ventilation). Several hundred additional milliliters of air were evacuated immediately following thoracostomy tube placement.

**Figure 3:**
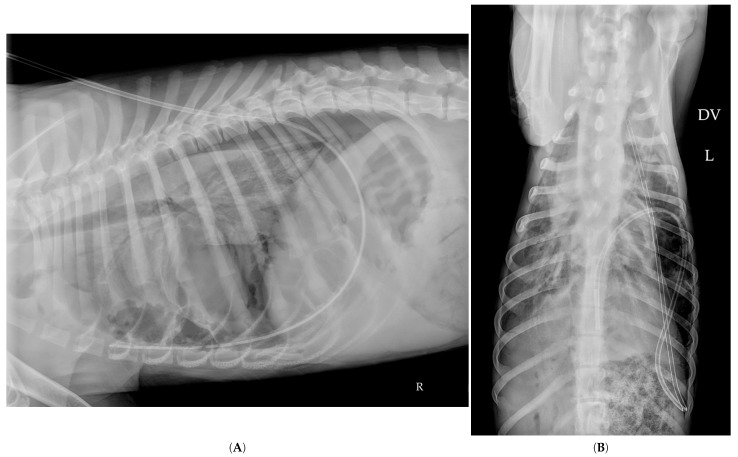
Right lateral (**A**) and dorsoventral (**B**) thoracic radiographs taken on Day 0, two hours after admission, following thoracostomy tube placement and evacuation of intrathoracic air. The previously identified air-filled cavity was no longer visible, indicating resolution of the compressive effect. A marked alveolar and interstitial opacity was observed throughout the lung parenchyma.

The follow-up radiograph taken after thoracostomy tube placement and evacuation of intrathoracic air confirmed the resolution of the compressive effect, with complete disappearance of the large air-filled cavity (Figure 3A,B). A marked alveolar and interstitial opacity was observed throughout the lung parenchyma. Although initially attributed to re-expansion pulmonary edema, this finding was retrospectively reinterpreted as passive lobar atelectasis (see Discussion (Section 3)).

### 2.10. Hospitalization Follow-Up

The decision was made not to use a Heimlich valve in order to quantify the amount of air evacuated through the thoracostomy tube. Thoracic aspirations were performed every 4 h on the day of admission, allowing for the collection of approximately 150 mL of slightly hemorrhagic fluid that was found to be moderately inflammatory on microscopic examination, without infectious organisms or neoplastic cells. No additional air was retrieved initially; however, at 4 a.m., 17 h after admission, the respiratory rate increased again, and 250 mL of air were evacuated through the thoracostomy tube.

The next morning, on Day 1, the dog’s general condition had significantly improved: the dog was standing, alert during walks, and had a very good appetite. Throughout the day, aspirations through the thoracostomy tube every 4 h yielded about 5 mL of fluid per session and only 10 mL of air over 24 h. On radiographs, the walls of the giant cavity were visible, but it no longer contained air. Several air-filled cavities persisted cranial to the heart, mostly visible on left lateral recumbency. The previously noted alveolar opacities, initially suspected to reflect re-expansion pulmonary edema, had substantially resolved, with the pulmonary fields appearing nearly normal.

On Days 2 and 3, mild pleural effusion and residual interstitial changes were still visible on thoracic radiographs. As the dog remained clinically normal and no further air accumulation was detected, it was hypothesized that the thoracostomy tube itself might be contributing to pleural irritation. Consequently, the tube was removed 72 h after admission.

**Figure 4:**
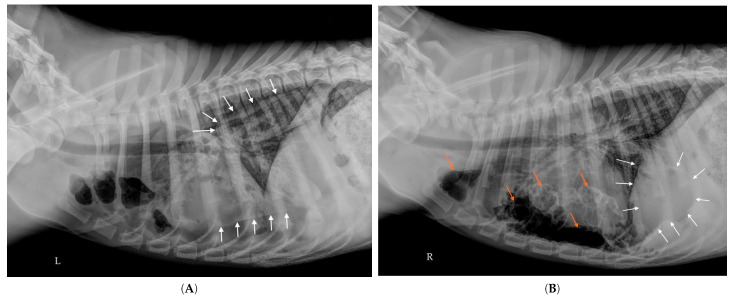
Lateral thoracic radiographs taken on Day 6, showing different images between the left lateral recumbency view (**A**) and the right lateral recumbency view (**B**) illustrating notable differences between projections. A large air-sac structure, likely corresponding to the wall of the giant air-filled cavity, was visible on both views (white arrows). The right lateral view showed numerous air-filled cavities (orange arrows) that were not visible on the left lateral view suggesting a gravitational redistribution.

On Day 6, radiographs in the left and right lateral recumbency provided noticeably different images (Figure 4A,B). On both projections, a large thin-walled sac, likely corresponding to the giant air-filled cavity, was visible. It appeared prominent on the left lateral view, but contained little to no air. Additionally, the left lateral recumbency view clearly showed four medium-sized air-filled cavities located cranially and ventrally, whereas the right lateral recumbency view suggested the presence of only two main air-filled cavities, associated with numerous smaller air-filled cavities that were not visible on the right lateral view.

On Day 7, despite the persistence of radiographic abnormalities, the dog was discharged from the hospital given the complete resolution of clinical signs.

**Figure 5:**
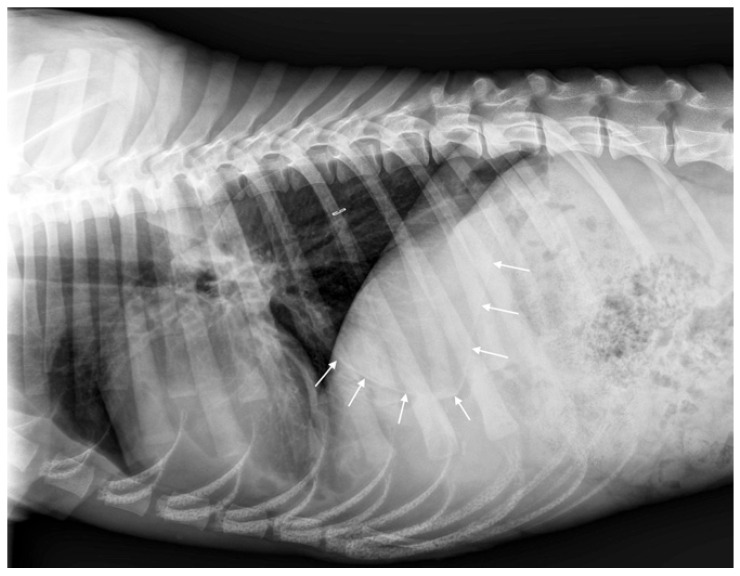
Lateral thoracic radiograph taken on Day 14, showing near-complete resolution of the initial pulmonary lesions. A large sac-like structure (white arrows), consistent with the persistence of the wall of the large air-filled cavity observed at admission, remains visible.

On Day 14, seven days after discharge, the dog was in excellent health. A follow-up thoracic radiograph performed by the referring veterinarian revealed near-complete resolution of the previously described pulmonary lesions. However, a large sac-like structure (white arrows) persisted in the caudal thorax, which was interpreted as the residual wall of the former giant air-filled cavity, now devoid of gas (Figure 5).

**Figure 6:**
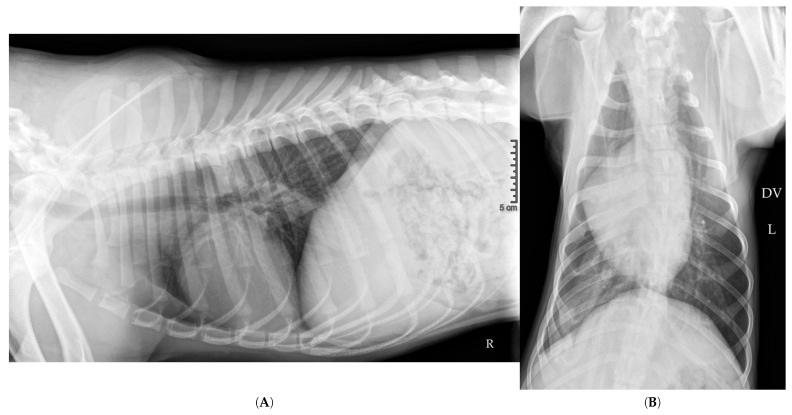
(**A**,**B**): Right lateral (**A**) and dorsoventral (**B**) thoracic radiographs taken on Day 56, showing near-complete resolution of the lesions, with no detectable air-filled cavities.

At two months post-admission, a final follow-up radiograph demonstrated complete radiographic resolution, with no visible residual air-filled cavities (Figure 6A,B). Clinical recovery was complete. The dog was adopted one month later and remained in excellent condition four months after the initial episode. Thoracic radiographs of its littermate, who had shared the same living environment, showed no abnormalities.

## 3. Discussion

This report describes the case of a 6-month-old Belgian Malinois with acute severe respiratory distress, in which thoracic imaging revealed a large intrathoracic air-filled cavity surrounded by numerous smaller satellite cavities. Prompt emergency management—comprising thoracocentesis followed by thoracostomy tube placement—resulted in rapid clinical improvement. A complete radiographic resolution was achieved within two months under conservative treatment.

### 3.1. Nature of Intrathoracic Air-Filled Cavities

In veterinary medicine as in human medicine, the identification of intrathoracic air-filled cavities presents diagnostic challenges due to the overlapping imaging characteristics of various entities. In the present case, three potential diagnoses were considered: pulmonary blebs, pulmonary bullae, and pneumatoceles. Differentiation among these relies on their anatomical location, underlying pathophysiology, and clinical evolution.

Pulmonary blebs are small accumulations of air located just beneath the visceral pleura. They typically appear as subpleural bubbles measuring less than 1 cm in diameter, most often found near the pulmonary apexes. Due to their extremely thin walls and peripheral location, blebs are particularly prone to rupture, which can lead to spontaneous pneumothorax in both humans and dogs [1,5].

Pulmonary bullae are larger air-filled cavities within the lung parenchyma, generally exceeding 1 cm in diameter. They result from the destruction of alveolar septa and the coalescence of adjacent alveoli. The walls of bullae consist of connective tissue when located within the lung parenchyma, or of visceral pleura when subpleural. Classically, bullae remain clinically silent until rupture occurs [1,6,7].

Pneumatoceles, also referred to as pulmonary pseudocysts, are thin-walled, air-filled cavities located within the lung parenchyma that typically develop in the context of acute pulmonary injury, including blunt chest trauma, barotrauma, ventilator-induced lung injury, or pulmonary infections [2,3]. In humans, these lesions are generally acquired, show a more dynamic evolution than bullae, and frequently resolve spontaneously under conservative management [3].

### 3.2. Distinction Between Blebs and Bullae

Distinguishing between blebs and bullae can be difficult based solely on radiography, especially since both types of lesions can coexist in the same individual [7,8]. This distinction can even be challenging on computed tomography (CT) [6,7,9,10,11]. Pulmonary lobectomy, precisely locating the lesion within the parenchyma, followed by histopathological examination, can help facilitate this distinction [8,12]. In light of the dog’s rapid clinical improvement under conservative management, advanced diagnostic modalities such as CT or surgical exploration were not pursued, given their invasiveness and cost, and the lack of indication in this clinical context.

Observing that bullae are often indistinguishable from blebs on both radiography and even CT, that the term “bullae” is often used interchangeably with “blebs” and even “bullous emphysema” in many publications [13,14,15], and considering that this distinction has no impact on clinical decision-making as the immediate management is common for all pneumothoraces, some authors question the relevance of distinguishing between the two in veterinary medicine [1,5]. In this discussion, blebs and bullae will be referred to as “bullae”.

### 3.3. Distinction Between Giant Bulla and Giant Pneumatocele

In the present case, the nature of the giant air-filled cavity remains a matter of debate: it may correspond either to a giant pulmonary bulla or to a giant pneumatocele [16]. Both hypotheses are supported by compelling arguments, which are summarized in Table 1. A comparative analysis of their respective epidemiology, pathophysiology, clinical evolution, and response to treatment may help guide the interpretation of the lesion.

#### 3.3.1. Epidemiology

The dog described herein was a 6-month-old neutered male Belgian Malinois.

Bullae are most often diagnosed following the occurrence of a spontaneous pneumothorax—a presentation that was notably absent in this case. Large case series investigating spontaneous pneumothorax in dogs—including 64 cases [12], 111 cases [17], and 110 cases [8]—report that this condition primarily affects medium to large, deep-chested dogs, most commonly large-breed dogs such as Siberian Huskies, Labrador Retrievers, Golden Retrievers, German Shepherds, Great Danes, and Boxers. In all three studies, the Siberian Husky was the most or second most represented breed. In these three studies, the median age was around 7 years [8,12,17], which is substantially older than the dog described in the present report. Interestingly, at just 6 months of age, this male dog, with a lean body condition, shares similarities with human patients with spontaneous pneumothorax, who are typically young, tall, and lean males [18]. Smoking is a well-documented predisposing factor in humans but is, of course, inapplicable to canine patients.

This young dog also shares similarities with human patients with pulmonary pneumatoceles, which are most frequently reported in infants, children, adolescents, and individuals with weakened immune systems [3]. In these populations, immature pulmonary architecture and increased susceptibility to alveolar injury may contribute to the development of pneumatoceles following acute insults such as infection, trauma, or barotrauma.

#### 3.3.2. Etiopathogenesis

No specific underlying cause was identified in this case. Given that pulmonary bullae typically evolve toward spontaneous pneumothorax over time, we initially investigated the most common causes of spontaneous pneumothorax in dogs. Because of the dog’s communal housing environment, CIRDC was considered; however, no clinical signs were consistent with this diagnosis: neither the dog nor its cage mates showed respiratory signs such as a loud, harsh cough suggestive of tracheitis. Parasitic etiologies, including *Angiostrongylus vasorum*, were ruled out, and *Dirofilaria immitis* is not endemic in our region. The dog’s young age and, more importantly, its complete clinical recovery made a neoplastic origin highly unlikely.

Two alternative hypotheses may explain the development of the observed pulmonary lesion: a congenital giant bulla or an acquired giant pneumatocele [19].

The formation of a giant bulla is generally a slow and progressive process, usually linked to congenital malformations, emphysema, or chronic alveolar wall destruction. In this young dog, a congenital origin remains plausible. The lesion may have been present but asymptomatic during the dog’s stay in the shelter, only becoming apparent after transfer to the rescue center, where closer monitoring facilitated detection.

The pathophysiological mechanisms leading to pneumatocele formation are well-documented in human medicine. Traumatic pneumatoceles typically occur in children and young adults following non-penetrating chest trauma, such as motor vehicle accidents or falls. These lesions result from a sudden increase in intra-alveolar pressure causing alveolar rupture and air dissection through the interstitium, forming a thin-walled cavity [20]. This mechanism also encompasses barotrauma and ventilator-induced lung injury, both of which can generate sufficient pressure gradients to induce alveolar rupture and subsequent pneumatocele formation. In the present case, recent stress—related to transportation, separation from littermates, and environmental change—may have induced transient hyperventilation, potentially contributing to alveolar overdistension. However, we acknowledge that this remains speculative and that, to our knowledge, hyperventilation alone has not been reported as a sufficient trigger for pneumatocele formation. It therefore appears more plausible that the dog sustained an unrecognized traumatic event or another form of pulmonary insult, which might have gone unnoticed.

#### 3.3.3. Clinical Presentation: Innumerable Air-Filled Cavities

The unusually high number of cavitary lesions in this dog represents a distinctive feature warranting closer comparison with both veterinary and human cases.

Pulmonary bullae are typically few in number. In the series by Howes et al. [17] including 111 cases and by Dickson et al. [8] including 110 cases, the median number of bullae or blebs identified during surgery was 1 (range: 1–6). However, in some descriptions, multiple bullae were present [1,12,21,22,23]. In the present case, the dog exhibited a strikingly high number of lesions, visible on radiographs as a complex network of cavities. Similar presentations have been reported in human patients, where large air-filled cavities may coexist with numerous smaller satellite lesions [24,25]. In dogs, two reports describe very young puppies with innumerable air-filled cavities [14,26]:

In one case reported by Tennant et al. [14], a 10-week-old Labrador Retriever puppy presented with respiratory distress; over twenty air-filled cavities ranging from 1 to 3 cm were identified on a lateral radiograph. Notably, no pneumothorax was associated with these cavities. The dog was euthanized, and the necropsy revealed a spectacular number of air-filled cavities distributed across all lung lobes.

In another case, reported by Brand et al. [26], an 8-week-old puppy weighing 2 kg presented with severe respiratory distress; radiography revealed innumerable coalescing pulmonary air-filled cavities. A tension pneumothorax was visible, likely secondary to the rupture of a pulmonary cavity prior to admission. During the first radiographic attempt, the puppy received cardiopulmonary resuscitation; therefore, it is not excluded that the pneumothorax was caused by the rupture of air-filled cavities during cardiac massage. The animal died after the radiographic examination. The necropsy confirmed the radiological lesions, revealing innumerable air-filled cavities up to 2 cm in diameter. Despite thorough post-mortem investigations, the origin of these lesions remained undetermined.

The extent of the lesions observed in the post-mortem examination of these two dogs did not seem compatible with survival and raises questions about the survival and even the complete recovery of the dog described in this report.

In both cases, the lesion was referred to as “congenital bullous emphysema”. However, we suggest that the authors misinterpreted the lesions as pulmonary bullae, while they may, in fact, have been describing extensive or coalescing pneumatoceles. As observed in the present case, the presence of multiple smaller satellite lesions supports the hypothesis of a dynamic and multifocal process, which is more consistent with the pathophysiology of pneumatoceles than with that of congenital bullae.

#### 3.3.4. Evolution

Since bullae are typically asymptomatic until they rupture and cause spontaneous pneumothorax, their treatment generally follows the same principles as for spontaneous pneumothorax. In veterinary medicine, surgical resection is often considered the treatment of choice, particularly in recurrent or non-resolving cases [1,8,12,17]. In the present case, surgery was not a viable option due to the number and distribution of the lesions: several dozen cavitary lesions were identified, diffusely affecting all pulmonary lobes.

Non-surgical management—including thoracocentesis followed by the placement of a thoracostomy tube—led to rapid clinical improvement. Air drainage ceased within 48 h, and the chest tube was removed on Day 3. The dog recovered uneventfully, with no recurrence of respiratory signs. Thoracic radiographs performed at follow-up showed complete resolution of the lesions. Such rapid and complete resolution is not a feature of true pulmonary bullae, which are generally stable or progressive and do not spontaneously regress. In contrast, pneumatoceles—both in human patients [3,27] and in dogs [2,28,29]—are known to regress within days to weeks once the precipitating insult has been removed. Spontaneous resolution without surgical intervention is often curative in these cases. The spontaneous regression and absence of recurrence following conservative treatment are features more consistent with a pneumatocele than with a bulla.

### 3.4. Originality of This Case

#### 3.4.1. Giant Air-Filled Cavity

In dogs, pulmonary bullae are typically small and preferentially located at the pulmonary apex [6]. However, their size can vary greatly, from a few millimeters to several centimeters. In humans, giant bullae are well described and are classically defined as air-filled cavities occupying more than one-third of a hemithorax [30]; but in dogs, we identified only two published cases of giant bullae.

Park et al. [31] reported a case of a giant pulmonary emphysematous cyst in a 12-year-old female Shih Tzu, incidentally discovered during preanesthetic thoracic imaging. CT confirmed a large, thin-walled air-filled cavity measuring 7 × 6 × 8 cm, occupying more than half of the left hemithorax, thus meeting the criteria for a giant bulla. The lesion was surgically removed via lobectomy of the left caudal lung lobe. Histopathological analysis revealed a well-differentiated cystic bronchioalveolar papillary carcinoma lining the internal surface of the cavity, raising the question of whether the bulla was primary or secondary to the tumor. No vascular or lymphatic invasion was detected, and follow-up CT scans up to 11 months later showed no recurrence or metastasis.

Roque et al. [32] described a second case in a 14-year-old mixed-breed female dog presenting with chronic cough, fatigue, and exercise intolerance. Thoracic radiographs and CT revealed a large emphysematous cavity in the left caudal lung lobe, measuring 8 × 7.5 × 3 cm, with thickened and irregular walls, and causing bronchial displacement and compression. The lesion fulfilled the definition of a giant bulla. Histopathology after thoracotomy revealed a bronchioalveolar adenocarcinoma with focal necrosis and dystrophic calcification. Despite initial postoperative improvement, the dog died four days later from cardiogenic pulmonary edema, unrelated to the surgical procedure.

In both cases, the giant bullae were considered secondary to neoplasia and presented in elderly dogs.

Pulmonary pneumatoceles—also referred to as traumatic pulmonary pseudocysts—are exceptionally rare in veterinary medicine. To date, only a limited number of cases have been reported in dogs and cats, most often in association with blunt thoracic trauma.

A case report by Mulholland and Keir [28] described a 1.5-year-old Labrador Retriever that developed multiple pulmonary pseudocysts following non-penetrating blunt thoracic trauma after being struck by a car. The dog presented with moderate respiratory signs, and thoracic radiographs revealed several well-circumscribed air-filled cavities distributed across the pulmonary fields. The lesions were confirmed on CT and interpreted as post-traumatic pseudocysts. Conservative management led to complete clinical recovery and full radiographic resolution within a few weeks. While the largest cavity measured 6.6 × 3.3 × 3.2 cm—making it a sizeable lesion—it did not meet the criteria for a “giant” pneumatocele as defined in human medicine (i.e., occupying more than one-third of the hemithorax). This case illustrates that post-traumatic pneumatoceles in dogs can be multiple, large, and fully reversible with supportive care alone, paralleling the presentation and outcome observed in the present case.

In a cross-sectional study involving 364 traumatized dogs undergoing thoracic CT, Bertolini et al. [29] identified pulmonary lacerations in 46 animals (12.6% prevalence). These lesions, described using a classification scheme analogous to that in human medicine, appeared as round or ovoid gas- or fluid-filled cavities within the lung parenchyma and were variably associated with rib fractures, pneumothorax, or contusions. The authors emphasized the discrepancy between CT and radiographic sensitivity: only 59% of lacerations seen on CT were detectable via thoracic radiography. Although the term “pulmonary pseudocyst” was not used by the authors, the imaging and clinical features described—including cavitary appearance, post-traumatic onset, and spontaneous resolution—are consistent with the pathophysiological and radiological definitions of pneumatoceles or traumatic pseudocysts as described in human medicine. The study further highlighted that younger and heavier dogs were significantly more likely to develop such lesions, likely due to biomechanical and anatomical factors. Most dogs were managed conservatively, with favorable outcomes, although two dogs required surgery for complications (abscess and lung collapse). No instance of an exceptionally large or giant pneumatocele was reported.

A recent retrospective study by González Montaño et al. [2] represents the most comprehensive veterinary case series to date on traumatic pulmonary pseudocysts (TPPs), detailing the clinical presentation, imaging features, treatment, and outcomes of nine dogs and two cats diagnosed by CT following blunt thoracic trauma. A total of 49 pseudocysts were identified, with a median of 4.5 lesions per animal. These lesions appeared as round to ovoid cavitary structures filled with gas or fluid, frequently associated with pneumothorax and pulmonary contusions. While thoracic radiographs detected TPPs in 64% of cases, the study confirmed the superior sensitivity of CT imaging for accurate identification. Most animals were successfully managed with non-invasive therapeutic approaches, and none died as a direct result of the pseudocysts. However, three patients required lung lobectomy due to persistent pneumothorax or concerns related to lesion size. One of the resected cavities measured 6.7 × 4.7 × 6.7 cm—again, not meeting the threshold for a “giant” pneumatocele. This study supports observations previously established in human medicine, showing that TPPs—although potentially large and multifocal—are often self-limiting and respond well to conservative therapy.

To the best of the authors’ knowledge, no previously documented case of a giant pneumatocele has been reported in the veterinary literature. In the present case, the air-filled cavity reached an estimated diameter of 14 cm at its widest point, making it the largest lesion of this type ever described in a peer-reviewed article involving a dog. It remains possible that similar lesions may have been reported in textbooks or on veterinary websites.

In humans, while giant pulmonary bullae are relatively frequently described, giant pneumatoceles are exceedingly rare, with only a few isolated cases reported [16,33,34,35,36,37,38,39]. Given its size, if the air-filled cavity observed in this case were indeed a pneumatocele, it would represent an exceptional finding in both human and veterinary medical literature.

#### 3.4.2. Confusion Between a Giant Bulla/Giant Pneumatocele and a Pneumothorax

In this case, the absence of pneumothorax on the initial presentation at Day -1 is noteworthy. It suggests that no bulla was ruptured at admission and that it was the volume of the giant air-filled cavity that caused the compression of the adjacent lung parenchyma, mimicking a tension pneumothorax. In most reported cases in veterinary medicine, bullae were asymptomatic until rupture, with spontaneous pneumothorax being the cause of symptoms. In this instance, the typical presentation of compressive pulmonary collapse and a quick reading of the radiograph—showing a wide area of hyperlucency—initially led to confusion with pneumothorax. The term “pneumothorax”, while etymologically defensible, is medically inappropriate, as the air was not freely present between the visceral and parietal pleurae but rather confined within a spherical cavity. The absence of a visible pulmonary border, the rounded morphology of the air-filled cavity, and the presence of similar cavities elsewhere all indicate that this was not a true pneumothorax [40].

The first description of a giant bulla compressing the lung parenchyma is attributed to Burke [41], who reported, in 1937, a case of “vanishing lungs” in a 35-year-old man with bronchial carcinoma. He described a giant bulla occupying four-fifths of the left hemithorax. However, we consider that Burke should not be credited with this discovery: similar cases had already been reported in the early 1930s [42,43,44], and even as early as the 1920s [45,46], as evidenced by the bibliography in the article of Haahti [42].

#### 3.4.3. Vanishing Lung Syndrome

In human medicine, despite the widespread availability of CT scanning, clinical case reports—including some recent ones—continue to describe confusion between a giant pulmonary bulla and a pneumothorax [47,48,49,50,51,52,53,54], without always mentioning the name associated with this syndrome: “vanishing lung syndrome”, referring to the title given by Burke to his article (“Vanishing lungs: a case report of bullous emphysema”) [41].

Vanishing lung syndrome refers to the presence of a giant pulmonary bulla compressing the adjacent lung parenchyma, thereby reducing functional lung volume [30,55]. The present case aligns closely with this definition. To the best of our knowledge, this is the first report in a dog of a giant cavitary causing pulmonary collapse that has been described under the term “vanishing lung syndrome”. We believe that Tennant et al. [14] briefly reported a similar case in 1987, although without explicitly referring to this term (see above). In human medicine, vanishing lung syndrome, as named, is frequently misinterpreted as pneumothorax on both radiographs and CT scans [50,56,57,58,59,60,61,62]; it is more common in young, thin smokers.

The term vanishing lung syndrome is traditionally reserved for cases involving giant bullae that prevent normal pulmonary expansion; it is not commonly used in reference to pneumatoceles. We identified two cases in the human medical literature in which giant pneumatoceles were initially misdiagnosed as pneumothorax [63,64]; however, neither of these cases made any reference to vanishing lung syndrome. In contrast, we found a unique clinical report by Hariri et al. [65], describing a young woman with a history of intravenous drug use who developed tricuspid valve endocarditis caused by *Staphylococcus aureus*, complicated by pulmonary septic emboli. The authors explicitly coined the term “pseudo-vanishing lung syndrome” to describe this unusual presentation, characterized by extensive bilateral pneumatoceles that radiographically mimicked vanishing lung syndrome, with massive air-filled cavities compressing the surrounding lung parenchyma. The clinical course was favorable, with almost complete radiographic resolution over several months. This case closely resembles the one we present here, as both patients exhibited a similar evolution. If the diagnosis of a pneumatocele is retained in our case, it could likewise be described as a pseudo-vanishing lung syndrome, or as a vanishing lung syndrome-like presentation.

#### 3.4.4. Percutaneous Drainage of the Giant Cavity

The objective of the thoracocenteses was to evacuate the air contained in the giant cavity, which involved puncturing it. Placing a thoracostomy tube into a giant bulla is considered an inadvertent procedure in human medicine, and this option is discouraged by some authors, who recommend performing a thoracic CT scan followed by resection of the bulla via thoracoscopy [40,48,54,66]. In the present case, severe respiratory distress posed an immediate, life-threatening emergency, which precluded both waiting for a CT scan and performing anesthesia. Surgery was not considered viable because of the extensive number and diffuse distribution of the lesions, with several dozen cavitary structures identified across all pulmonary lobes.

In human medicine, several case reports have documented the successful use of thoracic drainage in managing giant bullae associated with acute respiratory distress [67,68,69,70]. Percutaneous drainage of a well-circumscribed pneumatocele is a well-documented therapeutic option in the human pediatric literature [71,72,73,74,75,76].

#### 3.4.5. Re-Expansion Edema?

The radiograph taken after the chest drain placement showed an interstitial to alveolar pattern (Figure 3), which we initially identified as re-expansion pulmonary edema. Re-expansion pulmonary edema is an acute, potentially fatal lung lesion that occurs after the rapid re-inflation of a collapsed lung. It results from increased capillary permeability induced by mechanical forces and oxidative stress secondary to ischemia–reperfusion. Re-expansion edema following pleurocentesis has sometimes been described in human medicine after treatment for spontaneous pneumothorax [77,78,79,80,81]. The risk is estimated at 0.9% [78]. A unique case of re-expansion pulmonary edema following puncture of a giant bulla was described in human medicine [82].

In the present case, the dog did not exhibit any deterioration in respiratory status, and radiographic improvement was observed in the absence of specific treatment. The dog was not mechanically ventilated, and since spontaneous breathing is generally associated with slower re-expansion of collapsed lung tissue, a rapid re-expansion is less likely. Moreover, the cranial displacement of the diaphragm (Figure 3 compared to Figure 2) indicates a markedly reduced intrathoracic volume, further supporting the hypothesis of passive lobar atelectasis.

## 4. Conclusions

This article reports an atypical case of compressive pulmonary collapse caused by a giant air-filled pulmonary cavity, accompanied by innumerable air-filled cavities, in a 6-month-old Belgian Malinois puppy housed in a kennel and recently exposed to a stressful event. Based on the available epidemiological, clinical, and radiographic data, the differential diagnosis in this case lies between a congenital giant pulmonary bulla and an acquired giant pneumatocele. Several arguments can be made in support of each hypothesis. The large size of the lesion is compatible with a bulla; giant bullae have occasionally been reported in pediatric human patients, usually in association with underlying congenital or emphysematous conditions. However, numerous elements favor the diagnosis of a pneumatocele: the dog’s young age, the acute onset of respiratory signs following recent stress, the presence of multiple smaller satellite cavities, and—most notably—the rapid and complete resolution of all lesions under conservative treatment. Such a favorable and reversible course is exceptional in cases of pulmonary bullae, which in humans are usually stable or slowly progressive and rarely resolve spontaneously. In contrast, pneumatoceles—particularly in pediatric patients—are known to regress once the inciting cause (such as trauma, barotrauma, or infection) is resolved. While histopathological confirmation was not pursued, the convergence of epidemiological, radiographic, and clinical findings supports the interpretation of a giant pneumatocele rather than a congenital bulla.

This is the first veterinary case explicitly described as resembling vanishing lung syndrome.

## Figures and Tables

**Table 1:** Differential diagnosis between pulmonary bulla and pneumatocele in humans.

Feature	Pulmonary Bulla	Pneumatocele
Etiology	Congenital or acquired: associated with emphysema, smoking	Acquired: post-infectious, trauma, barotrauma (mechanical ventilation)
Age of Onset	Typically affects young thin adults, especially smokers	More common in infants, children, and young adults
Clinical Context	Often incidental; may present with spontaneous pneumothorax	Occurs after pneumonia, chest trauma, or mechanical ventilation
Clinical Evolution	Progressive; may remain stable or enlarge over time	Generally resolves spontaneously or with conservative treatment
Symptoms	Often asymptomatic until rupture; large bullae can cause dyspnea	May be asymptomatic or cause mild respiratory distress
Imaging: Number of Lesions	Usually solitary or few in number	Multiple; can be solitary or numerous
Imaging: Distribution of Lesions	Typically subpleural, especially in upper lobes	Variable; can be unilateral or bilateral, often in areas of prior infection or trauma
Imaging: Wall Characteristics	Very thin-walled, often imperceptible on imaging	Thin-walled, well-defined air-filled cavities
Imaging: Shape	Well-circumscribed, round	Round or oval
Imaging: Size	Typically > 1 cm; giant bullae may occupy more than 30% of hemithorax	Varies; can be small or large, sometimes termed “giant” if very large
Association with Pneumothorax	Frequent; rupture is a common cause of spontaneous pneumothorax	Can be associated, especially if ruptured
Response to Conservative Treatment	Rarely regresses; may require surgical intervention if symptomatic	Often resolves without intervention
Prognosis	Variable; depends on size, number, and associated complications	Generally favorable with resolution of underlying cause
Histopathology	Air-filled space lined by attenuated epithelium; associated with alveolar wall destruction	Air-filled cavity without epithelial lining; may show organizing fibrosis

## Data Availability

All data are available on request.
